# Artificial intelligence in chronic disease self-management: current applications and future directions

**DOI:** 10.3389/fpubh.2025.1689911

**Published:** 2025-11-20

**Authors:** Ying Du, Peng Yang, Yuntao Liu, Chunxia Deng, Xin Li

**Affiliations:** 1 Department of Health Management, Affiliated Renhe Hospital of China Three Gorges University, Yichang, Hubei, China; 2 Department of Anesthesiology, Affiliated Renhe Hospital of China Three Gorges University, Yichang, Hubei, China; 3 Department of Endocrinology, Affiliated Renhe Hospital of China Three Gorges University, Yichang, Hubei, China; 4 Department of General Surgery, Affiliated Renhe Hospital of China Three Gorges University, Yichang, Hubei, China; 5 Department of Nursing, Affiliated Renhe Hospital of China Three Gorges University, Yichang, Hubei, China

**Keywords:** artificial intelligence, chronic disease self-management, personalized interventions, predictive analytics, digital health platforms

## Abstract

**Objective:**

This study aims to summarize current applications of artificial intelligence (AI) for chronic disease self-management, critically appraise their effectiveness, and identify implementation challenges and future directions for research and clinical integration.

**Methods:**

A narrative literature review of peer-reviewed, English-language studies identified via PubMed, Web of Science, and Scopus was conducted, using combinations of “artificial intelligence,” “chronic disease,” “self-management,” “remote monitoring,” “predictive analytics,” “conversational agent,” and “mobile health.” Reference lists of key reviews were snowballed. We included studies that described or evaluated AI-enabled self-management tools or interventions for chronic conditions and excluded non-AI, acute-care, editorial, and non-human studies. Findings were synthesized thematically.

**Results:**

The literature consistently identifies four roles of AI in chronic care: (1) personalized decision support and treatment optimization; (2) continuous monitoring and risk prediction from patient-generated data; (3) conversational agents delivering education, adherence support, reminders, behavioral coaching, and mental-health support; and (4) AI-enabled Mobile health (mHealth) platforms that connect patients with clinicians and coordinate care. Recurrent challenges reported include data privacy and security risks, algorithmic bias and limited generalizability, interoperability and workflow-integration barriers, variable usability and sustained engagement (digital divide- inequalities in access to digital technologies and the internet, often influenced by age, income, or geography), and insufficient high-quality evidence on clinical effectiveness and cost-effectiveness.

**Conclusion:**

Future directions focus on developing more accurate, explainable, and trustworthy AI models, better clinical integration, leveraging advanced AI for engagement, rigorous evaluation, and addressing ethical and implementation barriers to realize AI’s full potential in empowering patients and improving chronic disease outcomes.

## 1 Introduction

Chronic diseases, such as diabetes, cardiovascular diseases, respiratory disorders, and mental health conditions, represent a significant global health burden, characterized by their long-term nature and the necessity for continuous management (1, 2). Effective self-management by patients is paramount to improving health outcomes, maintaining quality of life, and reducing healthcare costs (2, 3). Self-management involves a range of behaviors, including medication adherence, lifestyle modifications (diet, exercise), symptom monitoring, and active participation in treatment decisions (4, 5) However, supporting patients in consistently performing these complex tasks over a lifetime presents substantial challenges for traditional healthcare systems, often limited by resources and geographical barriers, particularly in rural or underserved areas (6, 7).

The rapid advancements in artificial intelligence (AI) and related digital health technologies, including mobile health (mHealth) and wearable devices, offer transformative potential to address these challenges and revolutionize chronic disease self-management (8–10). AI can process vast amounts of patient-generated data from various sources, such as Electronic Health Record (EHR)s, wearable sensors, and mobile applications, to provide personalized insights, predictive warnings, and interactive support, moving healthcare towards a more predictive, preventive, and personalized model (11). Unlike prior reviews that focus on single tools (e.g., chatbots) or single diseases (e.g., diabetes), our review takes a task-based, cross-modal perspective, mapping diverse AI technologies directly onto the core components of patient self-management. This approach offers a unified framework that clarifies both where AI has matured and where gaps remain. By examining diverse AI modalities and their implementation across various chronic conditions, this review seeks to provide a comprehensive overview of the evolving landscape and highlight areas requiring further research and development to fully realize AI’s potential in empowering patients and improving health outcomes.

## 2 Literature search and selection

This review followed a narrative approach. We searched PubMed, Web of Science, and Scopus for English-language studies published up to May 2025 using keyword combinations including “artificial intelligence,” “chronic disease,” “self-management,” “remote monitoring,” and “digital health.” Additional references were identified by screening citations in relevant reviews. We included studies that described or evaluated AI-enabled self-management tools for chronic conditions, while excluding non-AI, acute-care, non-human, and editorial/commentary articles. As this was a narrative review, no formal quality assessment or systematic synthesis (e.g., PRISMA flow diagram) was undertaken.

## 3 Current applications of AI in chronic disease self-management

The application of artificial intelligence in chronic disease self-management is multifaceted, leveraging various AI techniques to address different aspects of patient care and support. These applications can broadly be categorized by the type of AI technology employed or the specific function they serve within the self-management process. This section will delve into the current state of AI applications, examining their use in personalized interventions, predictive analytics, monitoring, and patient engagement tools like conversational agents—an AI-powered virtual assistant or chatbot that interacts with patients via text or speech to provide education, reminders, or coaching—drawing upon recent literature across different chronic conditions.

### 3.1 AI for personalized interventions and decision support

One of the most significant promises of AI in chronic disease management is its ability to enable personalized interventions and provide data-driven decision support, moving away from a “one-size-fits-all” approach (12). Machine learning (ML) algorithms analyze high-dimensional, longitudinal data to identify patterns and predict individual responses to treatments or lifestyle changes, thereby informing day-to-day self-management decisions. Diabetes exemplifies this precision approach: models integrating continuous glucose monitors (CGM), insulin pumps records, diet logs, and activity data generate tailored recommendations for insulin dosing, meal planning, and exercise with the goal of optimizing glycemic control (13–26). Diabetes education tools increasingly embed AI to personalize content and coaching, strengthening self-management skills (27, 28). Prospective system-validity studies demonstrate next-day hypoglycemia prediction from mobile/CGM data with random forest accuracy 0.814 (F1 = 0.812; sensitivity = 0.815; specificity = 0.824), outperforming alternative models (accuracy 0.65–0.80). Explainable Artificial Intelligence–driven Clinical Decision Support Systems (AI-CDSS) can make daily self-management more predictive and proactive by fusing data from smartphones, wearables, and CGMs (29), while “nurse-in-the-loop” predictive digital twin—a virtual, patient-specific model that simulates health status and treatment responses to support personalized care—strategies for Type 2 Diabetes (T2D) showcase how transfer-learned models can deliver individualized feedback aligned with clinical expertise (30). In oncology survivorship, emerging tools personalize surveillance and supportive-care recommendations (e.g., symptom triage, fatigue/exercise prescriptions, toxicity monitoring), aligning advice with individual risk profiles and preferences.

### 3.2 AI for monitoring and predictive analytics

A core contribution of AI to self-management is the ability to transform continuous, multimodal data streams—physiological signals from wearables and home devices, symptom reports, medication logs, and EHR histories into timely risk assessment and early-warning insights that patients and clinicians can act on between visits. Models trained on longitudinal data support near-real-time monitoring, short-horizon exacerbation prediction, and trend detection that trigger tailored advice or escalation pathways, thereby complementing decision support systems.

In cardiovascular disease, monitoring pipelines increasingly fuse signals such as heart rate dynamics and rhythm strips with clinical history to detect atrial fibrillation (AF) episodes, anticipate deterioration, and inform self-care prompts or remote reviews (31–35). These systems illustrate how passive sensing coupled with predictive analytics can shorten the time from signal to action in routine self-management.

The monitoring–prediction stack spans risk stratification, diagnostic augmentation, screening, and patient support. Explainable AI stratifies exacerbation risk from features such as smoking history, BMI, and symptom profiles (36). In specialist settings, adding explainable AI to pulmonologist PFT interpretation increased mean diagnostic accuracy versus pulmonologists alone—although a subset performed worse, underscoring the need for clinical oversight (36, 37). Imaging models provide staging and prognostic precision: CT deep learning in Chronic Obstructive Pulmonary Disease (COPD) Gene/ECLIPSE achieved ~50% exact GOLD staging and ~75% within one stage, predicted exacerbations and mortality, and reached AUC 0.89; PPV 0.847 for COPD identification in ECLIPSE; combining CT radiomics + demographics + spirometry further improved progression prediction over any single modality. At primary-care level, case-finding tools report usable operating points: a graph convolutional network achieved accuracy 0.77 (AUC 0.81) on weakly labeled CT; an AI “robot” questionnaire reached sensitivity 76.11%, specificity 84.76%, AUC 0.858; and a multi-instance learning classifier reported AUC 0.742 (38, 39).

Beyond cardiometabolic disease, monitoring and prediction are expanding across other conditions. In chronic kidney disease (CKD), longitudinal EMRs underpin AI models that predict progression trajectories to guide precision management (40, 41), with systems such as TrajVis translating model outputs into interpretable clinical insights (42). In addition, a comparative modeling study for early CKD identification trained a deep neural network (multilayer perceptron) on a 400-patient dataset (75/25 train–test split; McNemar test for model comparison) and reported 100% test accuracy, outperforming logistic regression (96%) and SVM (82%); the reported confusion matrix showed perfect classification of both CKD and non-CKD cases, underscoring potential while also highlighting the need for external validation and larger, multi-site cohorts (43). For end-stage kidney disease, proof-of-concept models suggest AI can help steer blood-pressure and volume strategies by learning from prior response, such as through artificial neural networks that predict session-specific outcomes and enable personalized adjustments to minimize intradialytic hypotension while optimizing fluid removal (44).

### 3.3 Conversational AI and intelligent coaching systems

These systems target engagement—personalized education, adherence support, and behavioral coaching—grounded in behavioral science and “nudge” principles. Unlike decision-support engines or passive risk models, engagement-focused systems emphasize human–AI dialogue and habit formation, with personalization and explainability as key levers (45–47).

Diabetes remains the most developed use case for AI-enabled engagement. mHealth apps increasingly integrate CGM/pump, diet, and activity data to deliver individualized education and real-time coaching around dosing, meals, and exercise. AI-driven education modules adapt content to literacy and learning needs (48–50), while nudge-based features encourage sustained lifestyle change (51, 52). Evidence maturity is mixed but encouraging: the breadth of tools reflects rapid diffusion in diabetes, and feasibility/acceptability is supported even in low-resource settings where peer-educator models are being tested to extend reach and cultural fit. At the same time, durable engagement and rigorous external validation remain necessary to translate personalization gains into consistent clinical improvements (53–55).

Conversational/ natural language processing (NLP) personalization can also improve engagement with diabetes education (mean engagement ratio 0.31 with personalization vs. 0.26 without) (56). In chronic limb-threatening ischemia (CLTI), AI/ML approaches aim to improve accurate diagnosis, outcome prediction, and identify disparities in treatment, highlighting the potential role for decision support in addressing inequities.

AI-powered digital pain coaches deliver individualized pacing, exercise, and cognitive-behavioral strategies via conversational interfaces. Prospective and multicenter studies report significant improvements in pain interference, physical function, and psychological distress over 12 weeks, supporting the role of tailored coaching in everyday self-management (57). These benefits underscore the potential of engagement-centric AI to shift outcomes even when pharmacologic or procedural options are limited.

Because anxiety, depression, and low mood commonly co-occur with chronic conditions, conversational agents increasingly provide personalized emotional support, self-monitoring, and triage. Early results suggest acceptability and user-reported benefit, but concerns around ethical safeguards, accuracy, and escalation pathways are prominent (58, 59), reinforcing the need for transparent reporting and integration with clinical oversight.

On the patient side, virtual agents co-designed with user’s support exacerbation action plans, mood management, and daily tasks with good acceptability and perceived usefulness (60, 61); at the sensing layer, smartphone auscultation and AI-augmented stethoscopes are being evaluated for wheeze/symptom recognition to enable home tele-monitoring (62, 63). Digital programs that embed algorithmic feedback improve quality of life and self-efficacy, although effects on healthcare utilization remain mixed (64).

### 3.4 AI in mHealth and digital health platforms

AI-enabled mHealth platforms coordinate information, decisions, and actions between patients and clinicians by fusing home-collected signals (wearables, sensors, symptom diaries) with EHR data, then routing triage, escalation, and follow-up through secure messaging, teleconsultation, and shared care plans. The aim is to close the “between-visit” gap with timely guidance, medication titration, and appointment or testing prompts while documenting actions back to the record. In heart failure (HF), platforms increasingly pair post-discharge risk models with symptom check-ins to trigger medication reconciliation, complication surveillance, rehabilitation/exercise pacing, and timely clinic contact. Evidence remains early-phase, but perioperative studies offer concrete operating points that motivate such transition workflows: ML can predict intraoperative hypotension up to 15 min in advance (AUC 0.95, sensitivity 88%, specificity 87%); continuous ward monitoring with gradient-boosted models has anticipated stage-2 AKI ~ 24 min before KDIGO criteria; and the PRODIGY score uses continuous capnography and oximetry to prospectively stratify risk of opioid-induced respiratory depression on general wards. At the same time, a pilot randomized evaluation of hypotension-prediction decision support did not reduce hypotension when alerts were frequently ignored, underscoring the need for robust workflow integration and escalation pathways as these tools move into the post-discharge transition context (65, 66).

### 3.5 Evidence maturity and gaps

**Figure 1:**
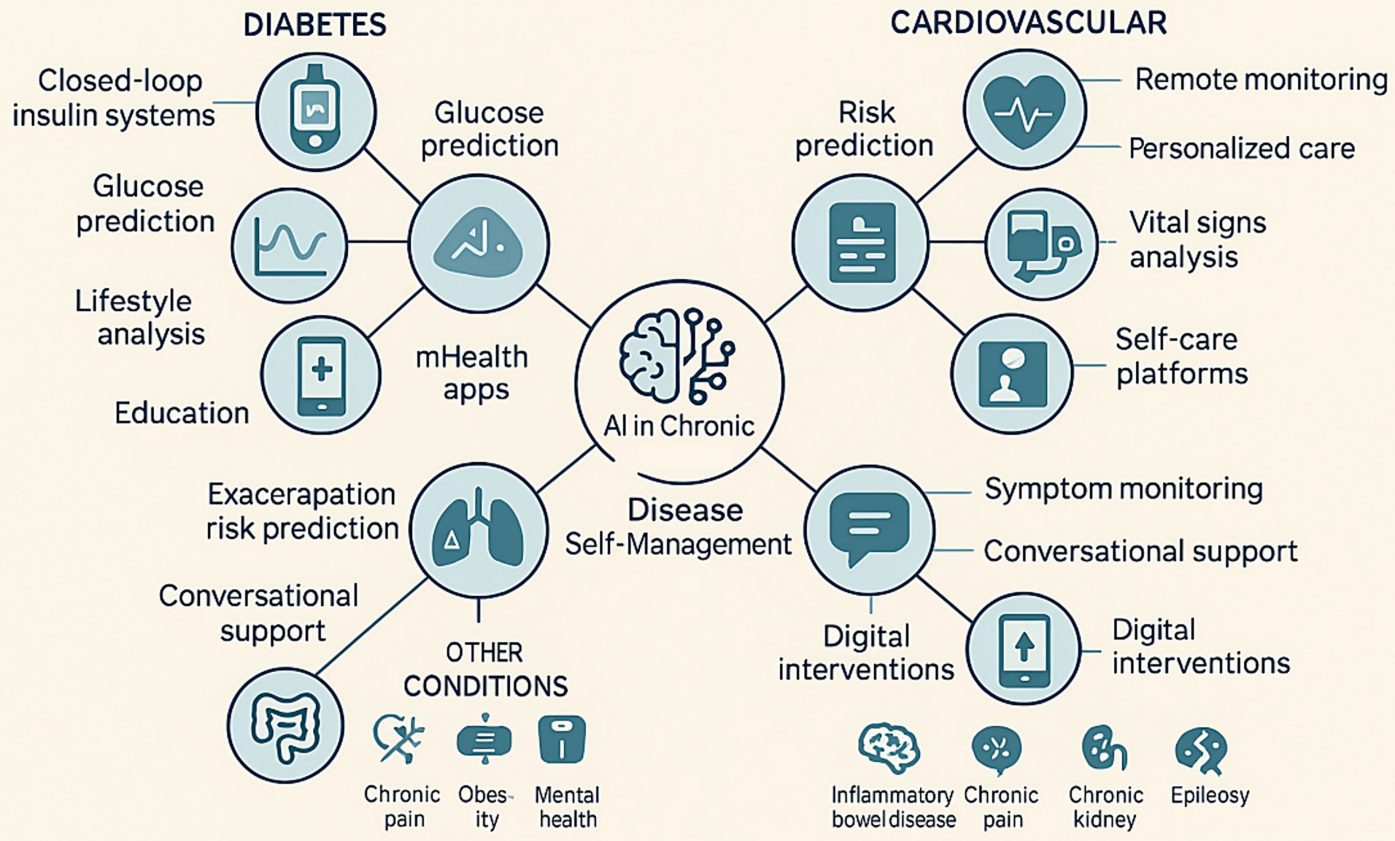
Examples of AI applications in chronic diseases. This figure illustrates various applications of AI in managing chronic conditions such as diabetes, cardiovascular diseases, and other chronic diseases (e.g., chronic pain, obesity, mental health). It highlights AI’s role in self-monitoring, telemedicine, risk prediction, and personalized treatment strategies.

Beyond functional categorizations, disease-specific manifestations of chronic conditions necessitate tailored AI approaches. This section explores these implementations across major chronic diseases, delineating how AI addresses distinct pathophysiological and self-management challenges (Table 1; Figure 1).

**Table 1:** AI applications in chronic disease self-management: key functions, technologies, and outcomes.

Chronic condition	Core AI functions	Key technologies/tools	Reported outcomes/advantages	References
Diabetes	Personalized insulin dosing; Glycemic prediction; Behavioral coaching	CGM + Closed-loop systems; AI mHealth apps; ML-based glucose prediction algorithms	Improved glycemic control; Reduced hypoglycemia events; Enhanced adherence to lifestyle modifications	(9, 19–24, 28, 32)
Cardiovascular diseases/heart failure	Remote deterioration detection; Risk stratification; Personalized care planning	Wearable sensors [ECG, blood pressure (BP)]; AI remote monitoring platforms; Predictive analytics models	Early detection of clinical deterioration; Reduced hospitalizations; Proactive intervention facilitation	(50, 67)
Chronic obstructive pulmonary disease (COPD)	Risk prediction; Exacerbation support; Symptom monitoring	Explainable AI (XAI) frameworks; Virtual conversational agents; Smartphone-based monitoring	Targeted screening; Improved self-efficacy during exacerbations; Enhanced quality of life	(34–37, 63)
Chronic pain	Activity-pain correlation analysis; Personalized behavioral guidance	AI-powered digital coaching platforms; Mobile apps with ML-based feedback systems	Reduced pain interference; Improved physical function; Decreased anxiety/depression	(70, 71)
Periodontitis	Oral hygiene adherence monitoring; Personalized feedback	AI-enabled toothbrush sensors; Targeted mHealth micromessages	Improved oral hygiene performance; Enhanced periodontal therapy outcomes	(33)
Other conditions^†^	Risk prediction; Symptom tracking; Personalized education	Chatbots (e.g., haemophilia); LLMs (e.g., ChatGPT); Predictive digital twins	Improved disease knowledge (haemophilia); Theoretical mental health support; Individualized management strategies	(12, 26, 45–47, 72, 73)

AI, Artificial Intelligence; BP, Blood Pressure; CGM, Continuous Glucose Monitoring; COPD, Chronic Obstructive Pulmonary Disease; ECG, Electrocardiogram; LLM, Large Language Model; ML, Machine Learning; mHealth, mobile Health; XAI, Explainable AI. Outcomes: Reflects outcomes reported in cited studies (e.g., “reduced hospitalizations” in CVD/HF; “improved oral hygiene” in periodontitis). ^†^Other Conditions: Includes haemophilia, mental health, hepatitis C, epilepsy, and inflammatory bowel disease where evidence is emerging but less established.

Across chronic conditions, the strongest maturation is seen in monitoring and predictive analytics, where longitudinal sensing and EHR data support near–real-time risk assessment and early-warning prompts. Feasibility and user acceptance are consistent in AF/CVD and COPD, and trajectory modelling in CKD provides actionable forecasts (42, 67, 68). However, The impact on service outcomes is heterogeneous and appears to depend strongly on patient engagement and workflow integration (43). For personalized decision support, promising signals come from diabetes (CGM/pump–integrated coaching, AI-CDSS, and nurse-in-the-loop “digital twin” strategies) and from HF care planning and titration. Yet broader clinical utility hinges on data quality, external validation, interpretability, and seamless integration into routines of care (12, 24, 69). Engagement-focused systems—conversational education, adherence support, and behavioral coaching—show improvements in patients-reported outcomes (PROs) in chronic pain and diabetes education, and are feasible even in low-resource settings (70, 71). At the same time, long-term retention is variable, effects on healthcare utilization remain mixed in respiratory disease programs, and ethical/accuracy questions persist for mental-health comorbidity agents.

Taken together, the field has progressed from feasibility toward prospective and real-world deployment in several niches, but generalizability and durability are the main bottlenecks. Priorities include: multi-site external validation and transparent reporting; pragmatic/hybrid-effectiveness evaluations with standardized PROs and economic endpoints; and design for sustained engagement and workflow interoperability so that model outputs translate into reliable, patient-centered action (32, 72, 73).

## 4 Challenges and considerations for AI in chronic disease self-management

Despite the transformative potential of AI in chronic disease self-management, significant challenges and considerations must be addressed to ensure its safe, effective, and equitable implementation. These challenges span technical, ethical, regulatory, and socio-behavioral domains (Table 2). A key concern is data privacy and security. AI systems for self-management often rely on collecting sensitive personal health data from various sources, including wearables, apps, and EHRs (6, 8). Robust cybersecurity measures and compliance with regulations are essential to maintain patient trust (6, 8). Ethical issues include algorithmic bias, transparency, and informed consent. Biased data may exacerbate disparities, while the “black box” nature of some AI models hinders trust among patients and clinicians. Enhancing transparency and ensuring meaningful informed consent remain priorities (8). Implementation barriers are also notable. Limited digital health literacy, especially among older adults and socioeconomically disadvantaged groups, and inequitable access to smartphones or internet connectivity restrict uptake (6, 74). Integrating AI into clinical workflows and EHRs poses technical and logistical hurdles, requiring interdisciplinary collaboration and training for health-care professionals. Importantly, even accurate models fail if alerts are ignored or escalation pathways are unclear, underscoring the need for workflow fit and human-in-the-loop oversight (43). Significant evidence gaps remain, particularly regarding long-term effectiveness, standardized outcome measures, and external validation across diverse populations. While monitoring and prediction tools show feasibility and patient-reported benefits, clinical outcomes remain heterogeneous and generalizability limited due to single-center or narrow cohorts. Large-scale, prospective validation with standardized outcomes is urgently needed. While engagement-focused AI demonstrates short-term improvements in PROs, challenges such as inconsistent retention and limited external validation highlight the need for further research. Similarly, mHealth platforms and telemonitoring improve adherence yet show mixed effects on hospitalization or long-term outcomes, with interoperability challenges limiting health-system deployment. Finally, In addition, ongoing regulatory uncertainty and concerns about patient trust present major barriers to widespread adoption. Frameworks for clinical AI remain in flux, complicating adoption (10). Sustained patient engagement requires building trust, as false alerts or over-reliance on automation can erode confidence (9). Addressing these multifaceted challenges demands coordinated efforts among researchers, developers, clinicians, policymakers, and patients.

**Table 2:** Cross-cutting challenges and solution requirements for AI in chronic disease self-management.

Challenge domain	Specific issues	Associated risks	Key solution requirements	References
Data privacy and security	Collection/storage of sensitive biometric, activity, and EHR data; Cross-platform data sharing	Patient data breaches; Non-compliance with regulations; Erosion of trust	Robust encryption (e.g., end-to-end); Strict adherence to GDPR/HIPAA; Decentralized data storage (e.g., federated learning)	(6, 8, 45, 69)
Algorithmic bias and transparency	Training data unrepresentativeness; “Black box” decision-making; Limited explainability	Perpetuation of health disparities; Clinician skepticism; Patient mistrust	Development of Explainable AI (XAI) frameworks; Diverse multi-center datasets; Algorithmic fairness audits	(8, 24, 42, 59, 68, 108)
Implementation barriers	Low digital literacy (e.g., elderly/low-SES groups); Limited tech access (rural/LMICs); Workflow integration difficulties	Digital divide exacerbation; Low adoption rates; Clinical workflow disruptions	Culturally adapted training programs; Low-cost/offline-capable devices; Interoperability standards (e.g., HL7 FHIR)	(6, 26, 32, 59, 74)
Evidence and regulatory gaps	Insufficient long-term efficacy data; Lack of standardized outcome measures; Evolving regulatory frameworks	Unproven clinical utility; Reimbursement uncertainties; Delayed market access	Large-scale RCTs with real-world endpoints; Consensus frameworks for AI validation; Adaptive regulatory pathways (e.g., FDA SaMD)	(10, 12, 66, 69)
Clinical integration and trust	False alerts (e.g., AF detection); Lack of clinician training; Patient over-reliance	Alert fatigue; Reduced confidence in AI; Undermined patient-clinician relationships	Hybrid “human-in-the-loop” oversight (e.g., nurse-led AI); Clinician education modules; Fail-safe mechanisms for critical alerts	(22, 24, 25, 66)

AF, Atrial Fibrillation; AI, Artificial Intelligence; EHR, Electronic Health Record; FDA, U. S. Food and Drug Administration; FHIR, Fast Healthcare Interoperability Resources; GDPR, General Data Protection Regulation; HIPAA, Health Insurance Portability and Accountability Act; HL7, Health Level Seven International; LMICs, Low- and Middle-Income Countries; RCT, Randomized Controlled Trial; SaMD, Software as a Medical Device; SES, Socioeconomic Status; XAI, Explainable AI.

In addition to these challenges, three barriers to broad adoption deserve emphasis. First, data integration and standardization remain formidable, as AI systems require multimodal, large-scale datasets combining imaging, EHR, and wearable signals. Heterogeneous data formats, inconsistent data quality, and siloed healthcare systems complicate the development of robust AI models, often requiring advanced data harmonization techniques and interoperable platforms to ensure compatibility across diverse data sources. For example, integrating continuous glucose monitoring data with EHRs for diabetes management demands standardized ontologies and secure data-sharing protocols to enable real-time, actionable insights (75). Second, regulatory clarity is evolving; notably, the U. S. Food and Drug Administration (FDA)’s December 2024 guidance on “Predetermined Change Control Plans” for continuously learning AI devices marks an important milestone. This guidance facilitates adaptive AI algorithms by allowing pre-approved modifications while ensuring safety and efficacy, yet uncertainties around reimbursement and liability persist, delaying integration into clinical practice (76). Harmonizing global regulatory frameworks remains critical to streamline deployment across jurisdictions (77). Third, ethical considerations are paramount: explainable AI is critical to foster clinician and patient trust, and physicians should play a leading role in driving AI innovation to ensure alignment with real-world clinical needs. Explainable AI models, such as those incorporating interpretable decision trees or attention mechanisms, enhance transparency by elucidating decision-making processes, thereby reducing skepticism among clinicians and patients (78). Moreover, physician-led innovation ensures AI tools address practical clinical challenges, such as optimizing chronic disease monitoring or personalizing treatment plans, by incorporating domain expertise into algorithm design and validation (79). Collaborative initiatives, such as clinician-researcher partnerships, are essential to align AI development with patient-centered care priorities (80).

## 5 Current AI use cases in cardiovascular medicine beyond self-management

AI applications extend beyond patient self-management to broader domains in cardiovascular medicine, including procedural, diagnostic, and efficiency areas. These use cases demonstrate AI’s versatility in addressing AF and related conditions, offering insights into potential integrations with self-management tools. This section outlines key examples, emphasizing their methodologies, findings, and implications, drawn from large-scale studies and clinical trials.

In AF screening and monitoring, consumer wearables have leveraged AI for large-scale, real-world detection. The Apple Heart Study, a prospective single-arm trial involving over 419,000 participants, utilized photoplethysmography (PPG) sensors on the Apple Watch combined with a ML algorithm to detect irregular pulses suggestive of AF. The algorithm achieved a positive predictive value of 84% for confirmed AF on subsequent ECG patches, with 34% of notified participants having AF episodes lasting ≥30 min, demonstrating high feasibility for opportunistic screening and user engagement, though limitations included underrepresentation of older adults (81). Similarly, the Fitbit Heart Study enrolled 455,699 participants and employed a PPG-based deep learning algorithm, reporting 98.7% sensitivity for AF episodes >30 min and a positive predictive value of 98% among those with irregular rhythms, underscoring cost-effective population-level monitoring with minimal false positives (82). The Samsung HEARTBEAT study, using Galaxy Watch devices, validated PPG algorithms against 12-lead ECGs, achieving 92.9% accuracy in AF detection over 14 days, highlighting usability for continuous monitoring in ambulatory settings (83). These studies illustrate AI’s potential to enhance early AF detection and burden quantification, bridging gaps in intermittent monitoring for chronic cardiovascular self-management.

Procedural challenges in AF ablation, such as prolonged ablation times (often exceeding 3–4 h), labor-intensive manual mapping, inconsistent lesion formation leading to incomplete transmurality, and difficulties in identifying extra-pulmonary vein (extra-PV) targets like rotors or focal drivers, have created opportunities for AI-driven solutions to streamline workflows, enhance precision, and improve outcomes (84, 85). These limitations in traditional approaches, which rely on operator experience and can result in recurrence rates of 20–40% in persistent AF, underscore the need for automated, data-driven tools that reduce variability and procedural duration while supporting personalized strategies (85, 86). For AI-assisted mapping and imaging, tools like Volta VX1 employ ML to analyze multipolar electrograms during AF ablation, identifying dispersion areas in real-time with high inter-operator agreement (kappa 0.85), improving procedural efficiency and reducing recurrence rates in persistent AF by targeting non-pulmonary vein drivers (87). CARTO AI, integrated into the CARTO electroanatomic mapping system, uses neural networks for automated annotation of complex fractionated atrial electrograms, facilitating faster substrate mapping and personalized ablation strategies, with studies showing reduced mapping time by 30% while maintaining accuracy comparable to expert manual review (88). Additionally, AI-guided MRI segmentation tools, such as deep learning-based convolutional neural networks for atrial scar and fibrosis delineation in late gadolinium-enhanced cardiac MRI, enable automated identification of extra-PV ablation targets with segmentation accuracy comparable to manual methods, reducing pre-procedural planning time and aiding in tailored lesion sets to minimize gaps and improve long-term success (89, 90). US2. AI echocardiography applies convolutional neural networks to automate full echocardiographic analysis, measuring parameters like ejection fraction and chamber dimensions with 95% agreement to expert readings, enabling rapid point-of-care diagnostics for HF and valvular disease in AF patients (91). These applications enhance precision in procedural interventions, potentially informing future AI-driven self-management by providing baseline data for personalized monitoring.

Efficiency tools further expand AI’s utility. AI risk scoring models, such as those predicting incident AF from clinical data and polygenic scores, integrate ML with EHRs to achieve AUCs of 0.85–0.90, outperforming traditional scores like CHARGE-AF by identifying high-risk individuals for preventive strategies (92). For ECG labeling, deep learning algorithms automate AF classification with >99% accuracy, reducing manual review time in large datasets and supporting scalable diagnostics (93). Prediction of AF from sinus rhythm ECGs uses convolutional neural networks to detect subtle features, predicting future AF with AUC 0.87 in cohorts like UK Biobank, aiding early intervention in at-risk populations (94). The TAILORED AF trial, a randomized controlled study, employed AI to guide real-time lesion delivery during ablation by mapping spatiotemporal dispersion, resulting in 74% freedom from AF at 12 months in the AI arm versus 58% in controls, demonstrating superior outcomes through adaptive, individualized procedures (95).

Collectively, these use cases exemplify AI’s clinical breadth, distinct from but complementary to patient-centered self-management, emphasizing the need for integration to optimize chronic disease outcomes.

## 6 Discussion

An important distinction that emerges from the reviewed literature is between early-phase feasibility or pilot studies and validated clinical applications. Many AI-enabled tools, such as conversational agents for patient engagement or predictive analytics for exacerbation risk, have primarily been evaluated in small, single-center feasibility trials or short-term pilots. These studies provide valuable proof-of-concept evidence and user-acceptability insights but do not establish clinical effectiveness. In contrast, validated clinical applications—those tested in large-scale, prospective, or real-world settings—remain relatively limited. For example, while some AI-supported decision support systems in diabetes and remote monitoring platforms in HF have undergone prospective evaluations and regulatory clearance, most other domains lack comparable validation (96, 97). This distinction underscores that the field, while promising, remains in a transitional stage between early feasibility studies and fully validated clinical applications. Feasibility evidence is encouraging, but widespread clinical adoption requires rigorous multi-site randomized trials, standardized outcome measures, and long-term effectiveness and cost-effectiveness data. Taken together, these observations underscore that while feasibility studies demonstrate promising signals. Validated applications, however, remain relatively scarce, underscoring the importance of conducting large-scale, multi-site clinical trials. In addition to these evidence-related challenges, regulatory oversight—particularly from the FDA—is playing an increasingly important role in shaping the clinical implementation of AI. The FDA regulates many AI systems under its Software as a Medical Device (SaMD) framework, requiring robust evaluation of safety, effectiveness, and quality assurance before approval (98). Recent guidance, including the introduction of “Predetermined Change Control Plans,” marks a pivotal step by allowing certain AI algorithms to adapt and update post-approval while maintaining oversight (99). Pilot initiatives such as the Digital Health Software Precertification Program also demonstrate the FDA’s intent to accelerate the evaluation process for digital health innovations (100). Nevertheless, evolving requirements and uncertainties regarding reimbursement, liability, and interoperability continue to create barriers for widespread adoption. Thus, FDA regulation acts as both a safeguard—ensuring patient safety and building trust—and a challenge, as stakeholders must continuously adapt to meet changing regulatory expectations.

The limitations of conventional monitoring strategies further illustrate this gap in clinical practice. For instance, intermittent approaches such as 24-h or 7-d ambulatory Electrocardiogram (ECG) recordings detect only a fraction of AF episodes, with reported sensitivities of approximately 16 and 42%, respectively, compared with implantable cardiac monitors that achieve up to 99% sensitivity in detecting AF episodes lasting ≥2 min, as demonstrated in a review of ambulatory ECG monitoring strategies where prolonged continuous monitoring significantly outperformed shorter intermittent recordings in identifying paroxysmal AF in patients with cryptogenic stroke or suspected arrhythmia (101). This limited sensitivity hampers timely diagnosis and accurate burden quantification, especially for patients with paroxysmal AF. AI-enabled wearable devices have the potential to overcome these limitations by providing continuous, unobtrusive monitoring in real-world settings. When coupled with advanced algorithms, these devices can enhance early AF detection, enable more precise assessment of arrhythmia burden, and support proactive intervention strategies. For example, large-scale screening studies like the Apple Heart Study utilized PPG-based algorithms with ML to detect irregular pulse suggestive of AF, achieving a positive predictive value of 84% for confirmed AF episodes and demonstrating high user engagement with over 400,000 participants, thereby highlighting the feasibility of AI-driven wearables for population-level AF detection and burden estimation in chronic cardiovascular self-management (81). Similarly, the Fitbit Heart Study employed PPG sensors integrated with deep learning models to identify AF, reporting a 98.7% sensitivity for AF episodes lasting >30 min and underscoring cost-effective, scalable alternatives to traditional monitoring (82). This context underscores how AI not only augments existing monitoring modalities but also addresses critical diagnostic blind spots inherent in conventional strategies.

Prior studies typically narrow to a single tool class (e.g., chatbots) or to a specific population/technology stack and largely inventory systems without linking them to the day-to-day work of self-management; chatbot reviews deem the evidence promising yet heterogeneous and note inconsistent technical reporting, limiting cross-disease comparability (102), while aging-focused surveys summarize ML/NLP advances but are not organized around patient tasks or mHealth/wearable workflows (103), and bibliometric overviews describe a fragmented field and call for implementation-relevant studies (104). In this review, we synthesize predictive analytics, continuous sensing/remote monitoring, conversational agents, and AI-enabled mHealth platforms and map them to core self-management tasks—personalized decision support; continuous monitoring and risk prediction; education/adherence/behavioral coaching; and patient–clinician care coordination—thereby clarifying where roles are maturing (e.g., monitoring/risk prediction) versus under-developed (e.g., sustained engagement and emotional support) and providing a cross-modal, task-anchored lens absent from chatbot-only work (102). We also reconcile mixed clinical signals by placing encouraging single-arm findings (e.g., an AI-powered digital pain coach over 12 weeks) alongside a null multicenter randomized controlled trial (RCT) (70, 105). Importantly, we connect patient-articulated priorities—personalization, emotional/social support, proactive monitoring, service integration, and ethics/governance (106)—to implementation pathways using NASSS and privacy-preserving infrastructures such as federated learning and blockchain. Unlike aging-focused reviews or platform papers that emphasize architectures without cross-disease appraisal (103, 107), our synthesis spans conditions and technologies yet remain organized by the work patients and clinicians actually perform. Collectively, this yields a pragmatic agenda—pragmatic/hybrid designs with standardized PROs and economic endpoints (70, 105), transparent technical reporting to enable comparability (102), and privacy-preserving data pipelines aligned with health-system integration (NASSS)—and, to our knowledge, the first cross-modal, task-based account of AI for chronic-disease self-management.

## 7 Future directions and opportunities

Looking ahead, the future of AI in chronic disease self-management holds immense potential, provided that current challenges are effectively addressed. Several key directions are emerging.

First, more trustworthy AI models are needed, with improvements in accuracy, reliability, and explainable AI (XAI) to enhance transparency and build trust among users and clinicians (12, 13). Ethical design principles must be embedded from the outset, actively working to mitigate bias and ensure equitable access (8). Second, integration into existing healthcare ecosystems is critical. Interoperable platforms should link patient-generated data from wearables and mHealth tools with EHRs and clinical workflows. Hybrid approaches such as “nurse-in-the-loop” models- a hydrid model where nurses oversee or validate the recommendations generated by AI systems, ensuring clinical appropriateness and patient safety-can combine AI efficiency with human oversight, ensuring care remains personalized and compassionate (20). Third, advancements in generative AI and NLP offer new opportunities to enhance patient engagement and education. Future conversational agents could become more empathetic, context-aware, and capable of delivering personalized self-management content, supporting adherence and sustained behavior change. Finally, rigorous evaluation and responsible implementation are essential. Large-scale RCTs and pragmatic trials are needed to establish effectiveness, cost-effectiveness, and long-term impact. Research should also address diverse patient populations, digital literacy gaps, and cultural adaptation. Clear regulatory pathways (e.g., recent FDA guidance on adaptive AI), along with interdisciplinary collaboration, will be crucial to ensure safe, scalable adoption.

## 8 Conclusion

Artificial intelligence is rapidly emerging as a powerful tool with the potential to fundamentally transform chronic disease self-management. By enabling personalized interventions, enhancing monitoring and predictive capabilities, and supporting patient engagement through conversational agents and digital platforms, AI provides innovative ways to help individuals manage complex, lifelong conditions.

Unlike prior reviews that typically focus on a single technology (e.g., chatbots) or a single disease (e.g., diabetes), this narrative review provides a task-oriented, cross-modal synthesis that integrates diverse AI modalities across multiple chronic conditions. This unique lens explicitly maps AI applications to the core self-management tasks of patients—personalized decision support, continuous monitoring and prediction, behavioral coaching, and patient–clinician care coordination—thereby offering a unifying framework absent from earlier work. In addition, our review incorporates the regulatory dimension, highlighting how evolving FDA guidance and other oversight frameworks critically shape the translation of AI into clinical practice, an aspect often underrepresented in the literature.

Current applications, particularly in diabetes and cardiovascular disease, show encouraging results such as improved treatment personalization, early detection of complications, and enhanced adherence. However, most evidence to date arises from feasibility or pilot studies, with validated clinical deployments in large-scale or real-world contexts still limited.

Bridging this gap requires rigorous multi-site trials, standardized outcome measures, and long-term effectiveness and cost-effectiveness evaluations. Only by generating robust clinical evidence and addressing challenges such as data privacy, algorithmic bias, and equitable access can AI move from promising prototypes to trustworthy, widely adopted solutions. Ultimately, by situating AI applications within both patient self-management tasks and real-world adoption pathways, this review contributes a novel and pragmatic roadmap for advancing AI toward reliable, patient-centered improvements in chronic disease outcomes.
